# Prevalence of associations among sarcopenia, obesity, and metabolic syndrome in Brazilian older adults

**DOI:** 10.3389/fmed.2023.1206545

**Published:** 2023-09-08

**Authors:** Luiz Carlos Holanda Torres Pinheiro, Marcelo Rossi, Carlos André Freitas dos Santos, Luis Vicente Franco Oliveira, Sergio Vencio, Rodolfo de Paula Vieira, Yara Juliano, Jane Armond, Carlos Hassel Mendes Silva, Adriano Luís Fonseca, Carolina Nunes França, André Luís Lacerda Bachi

**Affiliations:** ^1^Post-graduation Program in Health Science, Santo Amaro University (UNISA), São Paulo, Brazil; ^2^Discipline of Geriatrics and Gerontology, Department of Medicine, Paulista School of Medicine, Federal University of São Paulo (UNIFESP), São Paulo, Brazil; ^3^Postgraduate Program in Translational Medicine, Department of Medicine, Paulista School of Medicine, Federal University of São Paulo (UNIFESP), São Paulo, Brazil; ^4^Human Movement and Rehabilitation Post Graduation Program, Evangelical University of Goiás (UniEVANGELICA), Anápolis, Brazil; ^5^Institute of Pharmaceutical Sciences, Goiania, Brazil; ^6^Brazilian Institute of Teaching and Research in Pulmonary and Exercise Immunology (IBEPIPE), São José dos Campos, Brazil; ^7^Post-graduation Program in Science of Human and Rehabilitation, Federal University of São Paulo (UNIFESP), Santos, Brazil

**Keywords:** metabolic syndrome, sarcopenia, sarcopenic obesity, Conicity index, body mass index, obesity prevalence, aging

## Abstract

**Background:**

Although aging is a process associated with the development of obesity, metabolic syndrome (MetS), and sarcopenia, the prevalence of these conditions in older adults from São Paulo, Brazil, is unclear.

**Methods:**

Therefore, the current study aimed to investigate the prevalence of obesity, sarcopenia, and MetS, both separately and together, in a community-based sample of older adults from São Paulo, Brazil. Data from the medical records of 418 older adults of both genders, aged 60  years or older (mean age 69.3 ± 6.5  years), who were not physically active, were used to conduct this retrospective cross-sectional study. Anthropometric variables were used to determine both body mass index (BMI) and Conicity index (C index). Sarcopenia and MetS were defined according to the criteria of the European Working Group on Sarcopenia in Older People and by the Brazilian Society of Endocrinology and Metabolism, respectively.

**Results:**

Based on BMI, the group of older men (*n* = 91) showed a predominance of adequate weight (*n* = 49) and the group of older women (*n* = 327) showed a predominance of obesity (*n* = 181). In association with obesity, while only the group of older women presented with sarcopenia (*n* = 5), 52 older women and 9 older men presented with MetS, and two older women presented with sarcopenia + MetS [prevalence ratio = 0.0385, 95% CI (0.007;0.1924)]. Based on the C index, 58 older women and 11 older men presented with MetS, while the occurrence of sarcopenia or MetS + sarcopenia was found in 32 and 5 older women, respectively [prevalence ratio = 0.0910, 95% CI (0.037;0.2241)].

**Discussion:**

Our results suggest that obesity, as measured by BMI or the C Index, was more closely associated with the occurrence of MetS than sarcopenia, regardless of gender, and also that sarcopenic obesity was only found in the group of older women. Additionally, the prevalence ratio of obesity, sarcopenia, and MetS evidenced using the C index was 2.3 times higher than the values found using the BMI classification.

## 1. Introduction

Population aging is one of the most impactful global changes in different societies. For instance, in 1991, the number of people aged 60 and over represented 7.3% of the total population, whereas in 2025, this group will represent 15%. According to global projections, the number of older adults could reach 2 billion individuals in 2050, representing 21.5% of the world’s population (1, 2).

Among several characteristics, it is widely accepted that aging or senescence is a natural, dynamic, progressive, and therefore inevitable phenomenon in which morphological, functional, biochemical, and psychological alterations can be observed, resulting from the interaction of a series of variables, such as genetics, lifestyle, and diseases (3). With regard to lifestyle, there is convincing evidence that physical inactivity can promote metabolic syndrome (MetS), which predisposes to increased risk factors for the development of chronic diseases and comorbidities associated with aging, such as metabolic syndrome (4).

According to the World Health Organization (WHO), MetS can be fundamentally defined by clinical and laboratory data, and its worldwide prevalence is approximately 25 to 35% (5, 6). The literature points out that one of the factors that may potentiate the occurrence of MetS in older adults is the development of obesity associated with aging (7). The excessive increase in body weight due to the accumulation of fat in adipose tissue, a fact that characterizes obesity, varies between 20 and 40% in the older adult population, depending on the evaluation model used. It has been emphasized that the increase in the manifestation of obesity in older adults is associated, among other factors, with a significant reduction in the level of daily physical activity, which can even lead individuals to become sedentary (8).

In particular, the decline in physical activity observed in the older adult population is closely associated with the progressive loss of skeletal muscle mass, which can vary between 10 and 40%. Corroborating this information, studies have reported a 30 to 50% decrease in muscle mass in individuals between the ages of 40 and 80. This reduction is linked to a significant loss of functional capacity of approximately 3% per year after the age of 60. According to the literature, the reduction in skeletal muscle mass and loss of muscle strength (defined as dynapenia) associated with reduced physical mobility characterize the occurrence of the geriatric syndrome called sarcopenia. Sarcopenia is related to clinical outcomes such as loss of mobility, increased risk of falls, frailty syndrome, cardiovascular disease, neurodegenerative disease, and osteoporosis, and is also a predictor of mortality in older adults (9).

Recent epidemiologic studies have highlighted the possible coexistence of a sarcopenic condition in older adults with obesity, a situation called sarcopenic obesity (OS), which occurs mainly in individuals over 55 years of age (10).

Since obesity is a prominent factor in both MetS and sarcopenia in older adults, it is very important to correctly define its occurrence. According to the WHO, obesity can be defined as abnormal or excessive fat accumulation that poses a health risk. In addition to the BMI index, central obesity, which is common in older adults, can be estimated by the conicity index (C index), proposed by Valdez (11) in the early 1990s, as an indicator of adiposity and body fat distribution, especially in older adults (12).

Although it is possible to find reports on MetS and sarcopenia in the older adult population, studies aimed at comparing different models of obesity assessment and their association with the occurrence of MetS and sarcopenia in this population are still scarce in the literature. Therefore, the aim of the current study was, first, to evaluate the presence of obesity using two different models and, second, to correlate the presence of obesity with the manifestations of MetS and sarcopenia, either alone or together, in older adults.

## 2. Methods

### 2.1. Study population

This study has a retrospective cross-sectional design, based on the medical records of older adults attending the “Centro de Referência do Idoso – Casa do Idoso,” an institution belonging to the Municipal Social Assistance Office in São José dos Campos City, São Paulo, Brazil. The sample consisted of 418 individuals of both genders, aged 60 years and older at the time of data collection, without a diagnosis of chronic degenerative disease, type 1 diabetes, neoplasm, respiratory, renal, or liver disease, autoimmune, infectious, and/or neurological disease, and who were not engaged in any exercise training program.

It is paramount to mention that we obtained data from the population aged 60 years and older because, according to the Brazilian Ministry of Health, these individuals are considered older adults. Moreover, in the present study, we did not include older adults who reported regular engagement in any exercise training program, since it is widely known that the regular practice of exercise training is beneficial for everybody, particularly the older adult population, and can improve several physiological, metabolic, and physical characteristics. In addition, the older adults who were physically active reported regular engagement in a wide range of exercise training programs, which included types, intensities, and modalities that did not allow us to adequately separate them into one or even two or three groups.

**Figure 1:**
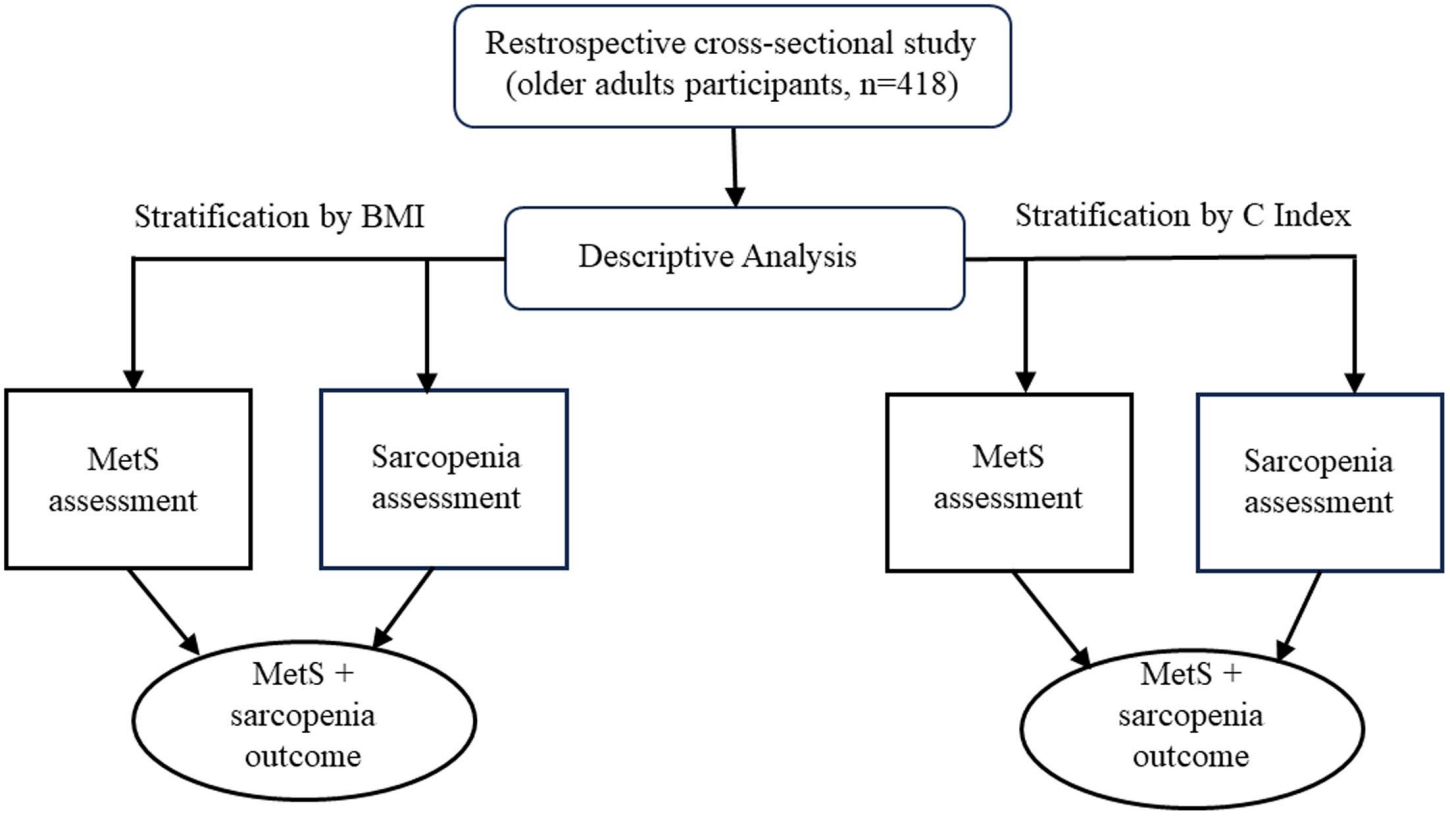
Flow diagram of the study design. BMI, Body Mass Index; C Index, Conicity Index; MetS, Metabolic Syndrome.

The primary endpoint was the diagnosis of MetS, and the secondary endpoint was sarcopenia. To assess the associations between MetS and sarcopenia, the BMI was measured at baseline, and the prevalence of the association between MetS and sarcopenia was determined for ranges of BMI. Figure 1 shows the analysis process performed.

### 2.2. Anthropometric measurements

The older adult participants were clinically evaluated by the same geriatrician responsible for each “Centro de Referência do Idoso – Casa do Idoso.” Data on the age, gender, race, body weight (kg), height (cm), and waist circumference (cm) of each subject were recorded in the initial database. Body composition was assessed using body mass index (BMI), which represents a diagnostic criterion for obesity, and muscle mass, which was defined using Lee’s equation (13), as shown below.

### 2.3. Body mass index calculation

Older adults were categorized by BMI, according to the stratification developed by Adams et al. (14) as follows: underweight = BMI < 18.5 kg/m^2^; adequate weight = BMI between 18.5 and 24.9 kg/m^2^; overweight = BMI between 25 and 29.9 kg/m^2^; and obesity = BMI > 30 kg/m^2^.

### 2.4. Conicity index calculation

The Conicity index (C index) was used to assess the visceral fat adipose mass as a measure of central adiposity obesity. This index was chosen because of the ease of interpretation of the values obtained. For instance, a C index of 1.25 indicates that the individual has a waist circumference 25% greater than the circumference of a cylinder with the same height, weight, waist circumference, and human body density. This index also offers the highest level of discrimination between MetS and sarcopenia.

The cut-off points were C Index values >1.25 for men and C Index >1.18 for women (15).

### 2.5. Assessment of metabolic syndrome

To determine the manifestation of metabolic syndrome (MetS), the criteria proposed by the Brazilian Society of Endocrinology and Metabolism were adopted (16), as also discussed in Freitas et al. (4).

### 2.6. Assessment of sarcopenia

The assessment of the occurrence of sarcopenia followed the criteria recently presented by the European Working Group on Sarcopenia in Older People (EWGSOP) (9). Muscle strength was determined using the handgrip test, with values < 29 kg for men and < 17 kg for women indicating low muscle strength. Muscle mass was estimated using appendicular skeletal muscle mass (aSMM) according to Lee’s equation (13), as described below:


$$\begin{array}{c} aSMM=0.244*bodyweight+7.8*height\\ +6.6*gender-0.098*age+\left(race-3.3\right) \end{array}$$

where body weight was measured in kilograms and height in meters; regarding gender, a value of 0 was used for women and 1 for men; regarding race, 0 was used for white people or Hispanics, 1.4 for African-Americans, and − 1.2 for Asian people. After applying the equation, the values obtained were divided by the square of the height (m^2^) of each subject to calculate the muscle mass index for each participant. The characterization of the presence of low muscle mass was defined when the values reached < 7 kg/m^2^ for men and < 5.5 kg/m^2^ for women.

### 2.7. Statistical analysis

Continuous variables were expressed as mean and standard deviation (X ± SD), and the number of subjects with MetS and sarcopenia was used to express prevalence data.

A McNemar’s test with continuity correction was used to test the hypothesis of an association between MetS and sarcopenia in the older adult population based on the BMI classification and C Index. In addition, both the BMI and C Index were used to compute the prevalence ratios (P_R_) for each group of participants (older women and older men), separated by their BMI and C Index values, showing an association with MetS and sarcopenia in comparison to those who did not show an association.

The statistical analysis was carried out using GraphPad Prism version 10.0 at a significance level of alpha = 5.0% (*p* < 0.05).

## 3. Results

Table 1 shows the anthropometric, physical, and clinical characteristics of the older adult participants in the present study, both pooled (total) and separated by gender.

**Table 1:** Anthropometric, body composition, and clinical characteristics data of the older adult participants in this study.

Groups	Participants			Variables
	Total *n* = 418 (100.0%)	Men *n* = 91 (21.7%)	Women *n* = 327 (78.3%)
Age (years)	69.3 ± 6.5	69.9 ± 6.4	69.1 ± 6.6
Weight (kg)	68.9 ± 13.7	77.0 ± 16.6	66.9 ± 12.1
Height (cm)	157 ± 9.0	167 ± 7.0	154 ± 6.0
Body Mass Index (BMI, kg/m^2^)	27.9 ± 4.7	27.4 ± 4.6	28.1 ± 4.8
Waist Size (cm)	92.97 ± 13.8	96.45 ± 13.9	92.12 ± 14.1
Conicity Index (CI)	1.29 ± 0.14	1.31 ± 0.12	1.28 ± 0.15
Appendicular Skeletal Muscle Mass (aSMM, kg/m^2^)	8.19 ± 1.55	10.13 ± 1.20	7.71 ± 1.21
Handgrip (kg)	23.6 ± 9.1	33.8 ± 11.8	20.8 ± 5.8
Clinical Aspects	27.9 ± 4.7	27.4 ± 4.6	28.1 ± 4.8
Type 2 Diabetes	98 (23.4%)	21 (23.08%)	77 (23.5%)
Hypertension	268 (64.1%)	53 (58.24%)	215 (65.8%)
Altered Cholesterol	184 (44.2%)	31 (34.06%)	153 (46.8%)

Values are presented as mean ± standard deviation (SD), and the clinical variables represent the number of subjects presenting with each clinical aspect.

Table 2 shows the number of participants with or without MetS and sarcopenia, both pooled (total) and separated by gender, in addition to their prevalence ratio. It was demonstrated that most of the subjects did not present with MetS and/or sarcopenia. It is worth mentioning that the absolute number of older women with MetS was higher than the value found in the group of older men, and the proportional occurrence of this syndrome in the group of older women was almost 1:4 (19.3%), whereas in the group of older men it was 1:6 (13.3%). In addition, because none of the older men presented with sarcopenia, the concomitant occurrence of MetS and sarcopenia was observed only in the group of older women, with a percentage of 1.8%. Interestingly, a McNemar’s test performed on the pooled subjects (total group) showed a significant value of *p*, which suggests an association between the occurrence of sarcopenia and MetS.

**Table 2:** The absolute number (*n*) and the percentage (%), total and categorized by gender, of subjects with metabolic syndrome (MetS), sarcopenia, or both (MetS + sarcopenia).

Groups	Total *n* (%)		Older men n (%)		Older women n (%)	
Clinical manifestations	*418* (100.0)		*91* (100.0)		*327* (100.0)	
	NO	YES	NO	YES	NO	YES
Sarcopenia	*371* (88.8%)	*47* (11.2%)	*91* (100%)	*0* (0.0%)	*280* (85.6%)	*47* (14.4%)
MetS	*342* (81.8%)	*7*6 (18.2%)	*78* (85.7%)	*13* (13.3%)	*264* (80.7%)	*63* (19.3%)
MetS + Sarcopenia	*412* (98.6%)	*6* (1.4%)	*91* (100%)	*0* (0.0%)	*321* (98.1%)	*6* (1.8%)
McNemar’s value of p*	*p* = 0.2944	*p* = 0.0116	*p* = 0.3560	–	*p* = 0.5201	*p* = 0.1527

The significant value of *p* was set at *p* < 0.05.

*McNemar’s test with continuity correction.

Table 3 presents the classification of the participants according to their BMI values, both pooled (total) and separated by gender. According to the values found, it is possible to demonstrate that the majority of older men were classified as having adequate weight, while older women were classified as obese. The table also shows the prevalence of MetS and sarcopenia in the older adult subjects categorized as underweight, adequate weight, or obese according to their BMI values. Regarding the group of older men, it was found that the participants with adequate weight or obesity had MetS, and the number of older adults with obesity with MetS was almost two times higher than those with adequate weight. As previously noted, none of these subjects presented with sarcopenia. However, in relation to the older women group, the occurrence of MetS was verified in all groups when separated by their nutritional status. It is important to point out that the number of older women with MetS increased concomitantly with the increase in BMI values; thus, the obese subgroup showed the highest number of individuals with MetS. Specifically, regarding the occurrence of sarcopenia, the adequate weight subgroup had the highest incidence of this clinical condition, followed by the underweight group and the obese group. Interestingly, the number of older women with MetS and sarcopenia was similar between the subgroups. Finally, in addition to the significant *p*-values shown in McNemar’s analysis, except in the group of older men with adequate weight, the prevalence ratios observed in the group of older women with obesity were 17- and 6-fold lower than those found in the underweight and adequate weight groups, respectively.

**Table 3:** The absolute number (*n*) and the percentage (%) of subjects with or without metabolic syndrome (MetS), sarcopenia, or both (MetS + sarcopenia), separated by gender and categorized by the BMI classification into underweight (< 18.5 kg/m2), adequate weight (between 18.5 and 24.9 kg/m2), and obese (> 30 kg/m2).

Groups	Volunteers (*n* = 418)					
	Underweight *n* (%)		Adequate weight *n* (%)		Obesity *n* (%)	
Clinical manifestations	Older men 4 (4.4)	Older women 24 (7.34)	Older men 49 (53.85)	Older women 122 (37.31)	Older men 38 (41.76)	Older women 181 (55.35)
Sarcopenia	*0* (0.0)	*16* (4.9)	*0* (0.0)	*26* (7.9)	*0* (0.0)	*5* (1.53)
MetS	*0* (0.0)	*2* (0.6)	*4* (4.4)	*9* (2.75)	*9* (9.9)	*52* (15.9)
MetS + Sarcopenia	*0* (0.0)	*2* (0.6)	*0* (0.0)	*2* (0.61)	*0* (0.0)	*2* (0.6)
None	*4* (100)	*8* (2.45)	*45* (49.45)	*90* (27.5)	*29* (31.8)	*126* (38.53)
McNemar’s value of p*	*––*	*p = 0.0022*	*p = 0.1336*	*p = 0.0058*	*p = 0.0077*	*p = 0.0001*
Prevalence Ratio [95% CI]	*––*	P_R_ = 1.5 [0.54;4.16]	*––*	P_R_ = 0.2343 [0.06;0.86]	*––*	P_R_ = 0.0385 [0.008;0.22]

The significant value of *p* was set at *p* < 0.05.

*McNemar’s test with continuity correction.

Table 4 shows the prevalence of MetS and sarcopenia in both groups classified as obese according to the C Index cut-off of 1.25 for men and 1.18 for women. It can be observed that only 11 older men presented with MetS, whereas 59 older women had been diagnosed with this syndrome. In addition, while 32 older women had sarcopenia, only five presented with both MetS and sarcopenia. Finally, the McNemar analysis showed significant associations between the outcomes in the two groups of subjects separated by the respective C Index cut-offs for men and women, and the prevalence ratio found in the group with older women was approximately 0.09.

**Table 4:** The absolute number (*n*) and percentage (%) of subjects, separated by gender, with C index values above the cut-offs of 1.25 for the older men group and above 1.18 for the older women group, and who also had or did not have metabolic syndrome (MetS), sarcopenia, or both (MetS + sarcopenia).

Groups	Volunteers (*n* = 418)	
	Older men *n* (%)	Older women *n* (%)
Clinical manifestations	C Index > 1.25 *n* = 68 (74.73)	C Index > 1.18 *n* = 284 (86.85)
Sarcopenia	*0* (0.0)	*32* (9.8)
MetS	*11* (12.1)	*59* (18.0)
MetS + Sarcopenia	*0* (0.0)	*5* (1.5)
None	*57* (62.6)	*193* (59.0)
McNemar’s value of p*	*p* = 0.0026	*p* = 0.0064
prevalence ratio	––	P_R_ = 0.0910 [95% CI (0.087; 0.2011)]

The significant value of *p* was set at *p* < 0.05.

*McNemar’s test with continuity correction.

**Figure 2:**
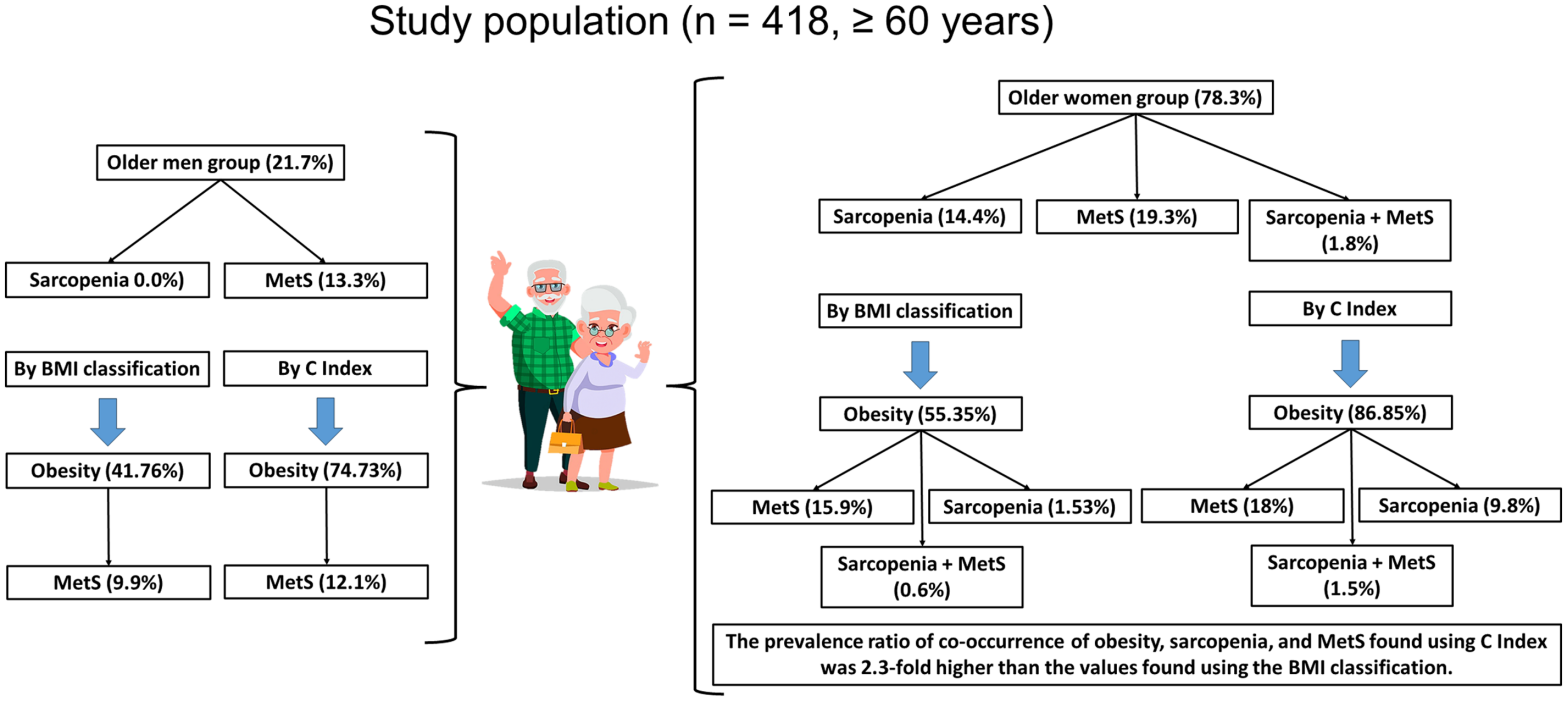
Summarized representation of the main results in this study. BMI, Body Mass Index; C Index, Conicity Index; MetS, Metabolic Syndrome.

Figure 2 summarizes the main data found in the study.

## 4. Discussion

In the present study, we demonstrated that when MetS was the primary outcome, among a total of 76 older adults who presented with this syndrome (Table 2), 63 older women and 13 older men also presented with obesity based on their BMI classification (Table 3). In addition, when sarcopenia was the primary outcome, it was found that, among the total of 47 older adults who had this clinical condition (Table 2), only five also had obesity based on BMI classification (Table 3), and they were found exclusively in the group of older women. Finally, it was demonstrated that the concomitant occurrence of MetS and sarcopenia was only observed in six older women, of whom two were underweight, two were of adequate weight, and two were obese (Table 3). Beyond these results, we also showed that, among the total subjects with obesity (*n* = 347), according to the C index (Table 4), the majority of individuals who also presented with MetS were in the group of older women (*n* = 59) compared to that of older men (*n* = 11). Moreover, in the same population that presented with obesity, the occurrence of sarcopenia was detected in 32 older women, while the concomitant occurrence of MetS and sarcopenia was detected in five older women. It is worth highlighting that the values of the prevalence ratio (P_R_) found in the group of older women classified as obese by the C index, who also had concomitant MetS and sarcopenia were 2.3 times higher than the values found in the group of older women classified as obese by the BMI values who also had concomitant MetS and sarcopenia. In addition, the significant results obtained in the McNemar test suggest the existence of a remarkable association between the occurrence of MetS and sarcopenia in the older adult population of participants in this study.

According to the WHO, obesity is a disease in which the accumulation of excess fat in the body can significantly affect human health. In fact, the prevalence of obesity worldwide is so high that the WHO considers it to be the global epidemic of the 21st century. Whether or not urgent action is taken to prevent and treat obesity, it is predicted that more than 50% of the world’s population will be obese in 2025. In this respect, the WHO reports that the occurrence of obesity can be defined through the BMI, and this index can also be used to estimate the prevalence of obesity in a population (17). However, it should be noted that although there is a good correlation between BMI and body fat mass, this index does not consider the variation in body fat distribution and may not correspond to the same degree of obesity or associated risks in different individuals and populations. Therefore, the WHO itself advises that BMI values should be interpreted with caution (18, 19).

Particularly in the context of clinical practice aimed at older adults, the evaluation of abdominal obesity by measuring waist circumference may be putatively considered a more important anthropometric measure than BMI to assess the risk of mortality. In agreement with the literature, the presence of visceral obesity is closely associated with the occurrence of dyslipidemia, arterial hypertension, endothelial dysfunction, polycystic ovary syndrome, coronary heart disease, and cerebral vascular disease. In addition, visceral obesity is also directly related to the development of insulin resistance and leads to the development of metabolic syndrome and death (20). In this sense, the use of the C index, as elaborated by Valdez et al. (11), is configured as an effective assessment of visceral obesity since it considers the distribution of central fat. It is paramount to point out that the C index evaluation not only takes into account weight, height, and waist circumference but is also based on the proposition that the accumulation of fat around the waist similar to “cone figure” in the human body (11, 21).

Pereira et al. (22) demonstrated that the C index showed a better association with the presence of MetS in both older men and older women compared to the results obtained with the BMI (22).

As appealing as genetic predisposition may be in determining the higher accumulation of fat in the body in some individuals, it is well known that this accumulation can also occur regardless of the individual’s genetics, and, in these situations, a lifestyle with an evident excess of energy intake associated with reduced physical activity leads to fat accumulation, especially in the abdominal region (18).

Reduced levels of daily physical activity or physical inactivity not only increase the risk of obesity but also the risk of sarcopenia (8, 9). With regard to sarcopenia, epidemiologic studies conducted in Brazil showed a prevalence of 14 and 16% in older men and older women, respectively (23). Furthermore, sarcopenia is closely associated with several clinical outcomes, such as loss of mobility, an increased risk of falls, frailty syndrome, cardiovascular disease, neurodegenerative disease, and osteoporosis, and is also a relevant predictor of mortality (9).

Among the various biological processes that induce sarcopenia, we can highlight the decrease in protein synthesis and/or increase in protein degradation, loss of neuromuscular integrity, and increased intramuscular fat content. In fact, the remarkable reduction in muscle mass observed in sarcopenia can be attributed to some factors, such as reduced growth hormone release and physical inactivity, which can influence a lower expression of key proteins involved in protein synthesis and, consequently, an increase in protein degradation, leading to muscle atrophy (9, 24).

The data obtained regarding the presence of sarcopenia in the studied individuals, particularly when stratified by the BMI and C indexes, revealed interesting results. In this sense, it is important to note that: (1) the occurrence of sarcopenia was only detected in the group of older women; (2) most of the older women who presented with sarcopenia were classified in the group as having adequate weight compared to the obese group, according to the BMI; and (3) the number of older women with obesity, according to the C index, who presented with sarcopenia was higher than the amount of participants found in the group of older women with adequate weight and obesity, both classified according to their BMI. Furthermore, the prevalence ratio [P_R_ = 0.0910 (95% CI 0.037, 0.2211)] observed in the group of older women with obesity, according to the C index, compared to the prevalence ratio [P_R_ = 0.0385 (95% CI [0.0077, 0.1924)] observed in the group of older women with obesity classified based on BMI, allows us to putatively suggest that the C index was more effective in defining the presence of sarcopenia in the older adult population participating in the study.

Specifically in relation to the association between sarcopenia and obesity, the literature shows the occurrence of a phenotype known as *sarcopenic obesity* (SO), especially during aging (10).

It is widely accepted that the manifestation of SO is related to genetic, physiological, and environmental factors. However, studies have demonstrated that some molecular mechanisms associated with SO are dependent on a dynamic balance between positive and negative mediating substances for muscle growth and that this balance impacts the maintenance of mass and skeletal muscle functions (25). Thus, it has been proposed that the occurrence of an imbalance in the following factors may lead to *sarcopenic obesity*: (i) primary metabolic abnormalities leading to increased systemic and muscular oxidative stress, with increased inflammation and insulin resistance; (ii) a consequent decrease in the hormonal balance, which may putatively stimulate a cascade of negative events, such as an increase in muscle catabolic potential; (iii) ectopic lipid deposition, which compromises protein turnover; (iv) mitochondrial dysfunction, causing an increase in oxidative stress, a reduction in the production of ATP, low production of muscle strength, and resistance to the prolonged exercise; (v) functionally altered muscle stem cells that can differentiate from adipocytes with a concomitant increase in inflammation; and (vi) physical inactivity, which is directly related to the control of positive energy balance, muscle oxidation, and protein turnover (26, 27).

Since OS translates as a phenotype caused by an imbalance of several factors, it is worth noting that its occurrence has a deleterious effect on the life of the individual, as it favors both the increased incidence of non-communicable chronic diseases and the low quality of life in these individuals (28). Thus, our observation that the C index was more sensitive than BMI in detecting the occurrence of OS may guide further studies to better define its prevalence.

Based on this information, there is no doubt that the manifestation of sarcopenia and MetS has a negative impact on the quality of life of the older adult population. Therefore, the concomitant manifestation of MetS and sarcopenia in the older adult population with obesity leads to an increased risk of the occurrence of adverse health events when compared to individuals who do not have both of these conditions or even those who have only MetS or sarcopenia (29). In this sense, in the meta-analytic study by Zhang and collaborators (30), around 35% of the non-obese individuals who presented with sarcopenia also manifested MetS, whereas only approximately 22% of the population studied without sarcopenia presented with MetS. In addition, the same authors also reported the existence of a significantly positive odds ratio (OR) between MetS and sarcopenia in the population studied, particularly in those with adequate weight (30).

Even though the above information demonstrates that an association between MetS and sarcopenia can be found in different populations, which could provide important data for medical assistance, a recent study highlighted that heterogeneous aspects of individuals, related to social, biological, and clinical characteristics, in conjunction with other aspects, such as the location and conditions in which the evaluations are conducted, represent key points in the decisions regarding the variables to be used in epidemiological studies since they undoubtedly impact the results obtained in a real context, particularly in relation to clinical practice (31). For instance, in the epidemiological studies that have provided data on the risk factors associated with the occurrence of both sarcopenia and MetS, it is possible to observe a large number of different factors reported in association with a diversity of criteria used, which inevitably generates greater heterogeneity in the results, which in turn can preclude the attainment of consistent conclusions (31–33).

Considering the above, we can emphasize that this study has some strengths related to the sample size and the robust statistical analysis. However, some limitations should be pointed out, such as: (i) the lack of body composition assessment using Dual-energy X-ray Absorptiometry (DXA), which is considered the gold standard for this measure, or even bioelectrical impedance analysis (BIA), which is an alternative and low-cost method often used in a large population; (ii) the lack of comparison of the data obtained in this study with other older adult groups who regularly engage in physical exercise; (iii) the lack of a dietary assessment, which could provide us with relevant information regarding the dietary habits of the older adult participants in this study; and (iv) the cross-sectional nature of this study, which does not allow us to establish cause-and-effect relationships between the data evaluated.

## 5. Conclusion

Based on the results obtained in the present study, we were able to demonstrate that the occurrence of MetS is higher in older adults who present with obesity, regardless of their gender and the use of the BMI or C index, which agrees with the literature. In addition, we also showed interesting results regarding the presence of sarcopenia, not only because t it was found exclusively in the group of older women but also by the fact that the highest number of participants with this clinical condition belonged to the group with adequate weight and not to the group with obesity, specifically when BMI was used. Additionally, taking into account the data obtained in the analysis of the prevalence ratios, as far as we were able to establish, this is the first study to demonstrate that the C index was more effective than the BMI in identifying the prevalence estimates of the occurrence of obesity and the clinical conditions assessed here (MetS and sarcopenia), both individually and in combination, in an older adult population. Finally, further studies are needed, both to confirm the results presented here and to better understand the use of the C index in relation to the prevalence of obesity, MetS, and sarcopenia in the older adult population.

## Data availability statement

The raw data supporting the conclusions of this article will be made available by the authors, without undue reservation.

## Ethics statement

Ethical review and approval was not required for the study on human participants in accordance with the local legislation and institutional requirements. Written informed consent from the patients/ participants was not required to participate in this study in accordance with the national legislation and the institutional requirements.

## Author contributions

AB conceived the study, analyzed the data, and wrote the first draft together with CS, SV, RP, and CF. LP, CS, AF, LO, and CF participated in the design and development of this study. LP, YJ, JA, and MR participated in the data analysis. All authors contributed to the article and approved the submitted version.

## Conflict of interest

The authors declare that the research was conducted in the absence of any commercial or financial relationships that could be construed as a potential conflict of interest.

## Publisher’s note

All claims expressed in this article are solely those of the authors and do not necessarily represent those of their affiliated organizations, or those of the publisher, the editors and the reviewers. Any product that may be evaluated in this article, or claim that may be made by its manufacturer, is not guaranteed or endorsed by the publisher.

## References

[1] Zaslavsky C Gus I. Idoso: doença cardíaca e comorbidade. Arq Bras Cardiol. (2002) 79:635–9. PMID: 12532249 10.1590/s0066-782x2002001500011

[2] Martín MAC Silleras BM Enciso LC Marcos SC de la Torre AM Río MPR. Changes in body composition in relation to the stage of demaletia in a group of institutionalized elderly. Nutr Hosp. (2013) 28:1093–01. 23889626 10.3305/nh.2013.28.4.6403

[3] SBGG. XIV Congresso Brasileiro de Geriatria e Gerontologia – GERON. Adriano Gordilho (coord.), Salvador – BA; Brazil, (2004).

[4] Freitas ED Fernandes AC Maledes LL Pimaleta AM Velásquez-Meléndez G. Metabolic syndrome: a review on diagnostic criteria. Rev Min Enferm. (2008) 12:403–1.

[5] Lyra R Oliveira M Lins D CavalcantI N Gross JL Maia FFR. Diretrizes Sociedade Brasileira de. Diabetes. (2003) 5:21–5.

[6] Haffner S Taegtmeyer H. Obesidade Epidêmica e Síndrome Metabólica. Circulation. (2003) 108:1541–5. doi: 10.1161/01.CIR.0000088845.17586.EC 14517149

[7] Mathus-Vliegen EM. Obesity and the elderly. J Clin Gastroenterol. (2012) 46:533–4. doi: 10.1097/MCG.0b013e31825692ce 22772735

[8] Jiang BC Villareal DT. Therapeutic and lifestyle approaches to obesity in older persons. Curr Opin Clin Nutr Metab Care. (2019) 22:30–6. doi: 10.1097/MCO.0000000000000520, PMID: 30346314 PMC6265116

[9] Cruz-Jentoft AJ Bahat G Bauer J Boirie Y Bruyère O Cederholm T. Sarcopenia: revised European consensus on definition and diagnosis. Age Ageing. (2019) 48:16–31. doi: 10.1093/ageing/afy169, PMID: 30312372 PMC6322506

[10] Merriwether EN Host HH Sinacore DR. Sarcopenic indices in community-dwelling older adults. J Geriatr Phys Ther. (2012) 35:118–5. doi: 10.1519/JPT.0b013e31823c4bef, PMID: 22166895 PMC3309150

[11] Valdez RA. Simple model-based index of abdominal adiposity. J Clin Epidemiol. (1991) 44:955–6. doi: 10.1016/0895-4356(91)90059-I, PMID: 1890438

[12] Saklayen MG. The global epidemic of the metabolic syndrome. Curr Hypertens Rep. (2018) 20:12. doi: 10.1007/s11906-018-0812-z, PMID: 29480368 PMC5866840

[13] Lee RC Wang Z Heo M Ross R Janssen I HeyMetSfield SB. Total-body skeletal muscle mass: development and cross validation of anthropometric prediction models. Am J Clin Nutr. (2000) 72:796–3. doi: 10.1093/ajcn/72.3.796, PMID: 10966902

[14] AdaMetS KF Schatzkin A Harris TB Kipnis V Mouw T Ballard-Barbash R. Overweight, obesity, and mortality in a large prospective cohort of persons 50 to 71 years old. N Engl J Med. (2006) 355:763–8. doi: 10.1056/NEJMoa055643, PMID: 16926275

[15] Pimaletel GMC Wanderley PTQC Tavares FCLP. Excesso de peso e índice de conicidade em idosos com diabetes mellitus. R Assoc bras Nutr. (2020) 11:59–71. doi: 10.47320/rasbran.2020.1662

[16] Sociedade Brasileira de Endocrinologia e Metabologia. Síndrome Metabólica, 2020, [acesso em 25abr2020] Disponível em: https://www.endocrino.org.br/a-sindrome-metabolica/

[17] WHO (2021). Available at: https://www.who.int/news-room/facts-in-pictures/detail/6-facts-on-obesity#:~:text=Body%20mass%20index%20(BMI)E28093,toormorethan2030.

[18] Afonso C IndexPN. Saúde, atividade física e peso corporal: contributo para o seu conhecimento numa amostra da população adulta Portuguesa. Mestrado em Saúde Pública. Faculdade de Medicina e Instituto de Ciências Biomédicas Abel Salazar, Universidade do Porto (1999).

[19] Abeso. Diretrizes brasileiras de obesidade 2016/ABESO. 4.ed – São Paulo, SP (2016). 1–8.

[20] Silveira RE Santos AS Sousa MC Monteiro TSA. Expenses related to hospital admissions for the elderly in Brazil: perspectives of a decade. Einstein. (2013) 11:514–09. doi: 10.1590/s1679-45082013000400019, PMID: 24488394 PMC4880392

[21] Milagres LC Martinho KO Milagres DC Franco FS Ribeiro AQ Novaes JF. Waist-to-height ratio and the conicity index are associated to cardiometabolic risk factors in the elderly population. Cien Saúde Colet. (2019) 24:1451–61. doi: 10.1590/1413-81232018244.12632017, PMID: 31066847

[22] Pereira MWM Arruda AL MetS L KmetS M KG D GSA A. Indicadores antropométricos associados a fatores de risco cardiovasculares em idosos. Rev Eletr Gest Saúde do Idoso. (2014) 5:3115–31.

[23] AlexandreTDSDuarteYAOSantosJLFWongRLebrãoML. Prevalence and associated factors of sarcopenia among elderly in Brazil: findings from the SABE study. J Nutr Health Aging. (2014) 18:284–09. doi: 10.1007/s12603-013-0413-0, PMID: 24626756

[24] Glass DJ. Signalling pathways that mediate skeletal muscle hypertrophy and atrophy. Nat Cell Biol. (2003) 5:87–90. doi: 10.1038/ncb0203-87 12563267

[25] Lozano-Montoya I Correa-Pérez A Abraha I Soiza RL Cherubini A O'Mahony D. Nonpharmacological interventions to treat physical frailty and sarcopenia in older patients: a systematic overview - the SENATOR project ONTOP series. Clin Interv Aging. (2017) 12:721–09. doi: 10.2147/CIA.S132496, PMID: 28490866 PMC5413484

[26] Kwon H Pessin JE. Adipokines mediate inflammation and insulin resistance. Front Endocrinol (Lausanne). (2013) 4:71. doi: 10.3389/fendo.2013.00071 23781214 PMC3679475

[27] Trouwborst I Verreijen A Memelink R Massanet P Boirie Y Weijs P. Exercise and nutrition strategies to counteract Sarcopenic obesity. Nutrients. (2018) 10:605. doi: 10.3390/nu10050605, PMID: 29757230 PMC5986485

[28] Zamboni M Mazzali G Fantin F Rossi A Di Francesco V. Sarcopenic obesity: a new category of obesity in the elderly. Nutr Metab Cardiovasc Dis. (2008) 18:388–5. doi: 10.1016/j.numecd.2007.10.002, PMID: 18395429

[29] Rubio-Ruiz ME Guarner-Lans V Pérez-Torres I Soto ME. Mechanisms underlying metabolic syndrome-related sarcopenia and possible therapeutic measures. Int J Mol Sci. (2019) 20:647. doi: 10.3390/ijMetS20030647, PMID: 30717377 PMC6387003

[30] Zhang H Lin S Gao T Zhong F Cai J Sun Y. Association between sarcopenia and metabolic syndrome in middle-aged and older non-obese adults: a systematic review and Meta-analysis. Nutrients. (2018) 10:364. doi: 10.3390/nu10030364, PMID: 29547573 PMC5872782

[31] Zupo R Moroni A Castellana F Gaspari C Catino F Lampignano L. A machine-learning approach to target clinical and biological features associated with sarcopenia: findings from northern and southern Italian aging populations. Meta. (2023) 13:565. doi: 10.3390/metabo13040565, PMID: 37110223 PMC10142879

[32] Tan A Thomas RL Campbell MD Prior SL Bracken RM Churm R. Effects of exercise training on metabolic syndrome risk factors in post-menopausal women – a systematic review and meta-analysis of randomized controlled trials. Clin Nutr. (2023) 42:337–1. doi: 10.1016/j.clnu.2023.01.008, PMID: 36736057

[33] Nishikawa H Asai A Fukunishi S Nishiguchi S Higuchi K. Metabolic syndrome and sarcopenia. Nutrients. (2021) 13:3519. doi: 10.3390/nu13103519, PMID: 34684520 PMC8541622

